# The Duty of the Dentist in Artificial Restoration, in Cases Where the Choice Lies between Removable Bridge, Fixed Bridge, or Partial Plate

**Published:** 1916-04

**Authors:** George H. Wilson


### THE DUTY OF THE DENTIST IN ARTIFICIAL RESTORATION, IN CASES WHERE THE CHOICE LIES BETWEEN REMOVABLE BRIDGE, FIXED BRIDGE, OR PARTIAL PLATE.

BY GEORGE H. WILSON, D.D.S.

"The duty of the dentist" opens up the moral or ethical phase of the means for the restoration in a partial loss of the teeth. The consideration of this phase of the subject may be classified under the headings:

First. What will best serve the health and life of the patient?

Second. Which method does the specific case economically justify?

Third. The qualifications and limitations of the dentist.

Before discussing these three propositions it is well to state that which we all consider a truism, that is, that the loss of even a single tooth is a loss that can never be fully restored. However, the conditions are such today that the loss of one or more teeth by accident or for health considerations is unavoidable. Therefore the great profession of dentistry has been evolved and is now developing in its most important field of usefulness, namely, the prevention of the loss of the teeth and their normal functions. Nevertheless, restorative dentistry will long be the engrossing part of the profession.

DEFINITIONS.

A fixed bridge is one spanning an edentulous space and is permanently attached to abutment teeth.

A removable bridge is one that has abutments fixed into or onto natural teeth, but the body of the bridge is removable. These bridges may have the body suspended between the abutments or the body may be partly supported by the gum tissue, a sort of pontoon bridge.

A plate bridge is one that is primarily a plate and the large part of its support is upon gum tissue. It is anchored into or onto one or more fixed abutments. These plate bridges may be either fixed or removable.

A partial plate is one supported by the gum tissue and retained by "suction," adaptation or clasps.

It is the conviction of the essayist that the consensus of opinion of all of the members of both the dental and medical professions, who are qualified to express mature judgment, is that the large fixed bridge is an abomination that should no longer be tolerated; and that the further insertion of them should be condemned. There seems to be a divided opinion regarding the advisability of small fixed bridges, as for instance, where one end of the bridge is fixed and the other is supported but not fixed. It is these small cases of one or possibly two teeth in a space that confronts every dentist and demands his professional opinion as to the best method of restoration. They should always be considered from the standpoints of health, economics and esthetics. No matter if a man's practice is limited to a special class of work, his mind should be open and broad enough so that he can render an unbiased opinion. The best judgment of the dentist may not meet with the approval of the patient, when the dentist should carefully weigh the patient's objections, and if erroneous, so acquaint the patient. At all times he should strive to give the objections a just valuation. It is regrettable that the dentist is too apt to be a faddist rather than a competent and fearless judge of the illusions of the day. That admonition of Pope's:

> "Be not the first by whom the new are tried;
> Nor yet the last to lay the old aside,"

is a sound principle for any professional man.

It is the duty of a professional man to arrive at a well matured opinion upon all professional subjects, and side issues that are cognate, in advance of the public, and use such acumen for the benefit of that portion of the public dependent upon him.

Note well this dogmatic statement: In this day of civilization and science, teeth (including artificial ones) are not essential; but both natural and artificial teeth may become a menace to health and life. However, both natural and properly constructed artificial teeth are of great economic value for they save much time in insalivating the food and preparing it for deglutition. They are also much prized because of the part they play in cosmetics. If these statements are accepted as truth (they are both logical and scientific) then the men who place such a high vital value upon teeth are open to the suspicion that they are not following the latter part of Pope's admonition, or, that they are actuated by commercialism.

These thoughts are suggested as an aid in solving the problems involved in the first of the three ethical propositions; that is, "What will best serve the health and life of the patient?"

In discussing the second proposition the professional man should not use the word economic in the sense of cheap or of smallness in price. It should be considered in the sense of efficiency for both service and justifiable expense.

Service efficiency demands for each case a relative knowledge of the comparative value of the various methods of restoration and a careful professional investigation of the present and future health conditions of the tissues and organs involved.

For the past thirty-five years metal crowns and fixed bridges have been a very important factor in restorative dentistry. They have been extensively used both skillfully and unskillfully, so that experience is matured; and, a just valuation may be placed upon the practice and various methods used. It is a rational conclusion to consider that shell crowns and fixed bridges have and may serve a good and justifiable purpose, but it is a safe guess that the large majority of their insertions have been questionable and that very many cases have been a menace to the health of the wearer and to the comfort of his friends; also, it is possible and quite probable that in some cases they were the main contributing cause of death. However, when a method, as practiced, is so severely condemned by competent judges the conscientious practitioner will rarely use it, and then only after careful consideration.

A partial loss of the dental organs may be restored by one of the three recognized methods, namely—fixed bridge, removable bridge or plate. The fixed bridge has the merits of comparative ease of construction and insertion, and, of security of retention. It has the demerits of being the most unsanitary of the three methods, difficult of proper repair when broken, and the most deficient in esthetic possibilities. The removable bridge has the merits of being more easily repaired, less unsanitary and has more esthetic possibilities than the fixed bridge. It has the demerits of being more complicated, consequently requires a higher degree of manipulative ability for its construction, and also is more fragile than the fixed variety. The partial plate has the merits of being the most sanitary of the three methods, of greater simplicity of construction, of greater esthetic possibilities, and is easily repaired. The demerits are that all of the pressure of mastication is upon the soft tissues, therefor may cause irritation, and is more bulky than the bridge. Some dentists are prejudiced against the clasping methods. This is unfortunate for undoubtedly more disintegration of tooth substance takes place in the recognized methods of properly constructed bridge work, either kind, than will ever take place under properly constructed clasp plates. Undoubtedly more decay and foulness occur under illy constructed bridges than under illy constructed partial plates. It is true that partial plates are not as sanitary as is desirable, but they are more sanitary than any bridge (either kind) that could have been placed in the specific case. These prejudiced dentists are often too hasty in their condemnation of some of the artificial appliances upon which they pass judgment; for often the deplorable condition they are contemplating is primarily the criminal negligence of the patient, and secondarily an error of judgment, upon the part of the dentist, in inserting anything in the obvious environment. At other times it is the result of an error in the scheme of construction, or the conditions have so changed that the appliance has served its day.

Having determined the desirability of and the kind of an appliance suitable for the specific case it is necessary to consider whether the expense is justifiable or not. We are informed that fifty per cent of the earners of this country do not have, to exceed, $50 per month; that thirty-five per cent more do not exceed $80 per month; that twelve per cent have between $80 and $300 per month, and that three per cent only exceed $300 per month. Another way of expressing this economic condition of society is in the statement that the submerged ten per cent of the population is incapable of supporting itself, that the great middle class, comprising eighty per cent of the people, are capable and will support themselves, while the affluent ten per cent must bear the burden of the unfortunate ten per cent.

With these facts before us it is evident that inexpensive dental methods are an imperative necessity, and that an army of dentists is required for this worthy middle class, while only a company of dentists can be employed by the less than three per cent that are justified in paying more than a nominal fee for professional services. It is essential that a dentist shall have his fees and methods (not quality) in keeping with the financial condition of the people whom he serves. Therefore, the very expensive methods of removable bridge work must be limited to a few patients and practitioners. Whereas the fixed bridge is questionable and rarely justifiable in any case, there is no choice in a large majority of cases, but to insert a plate if anything is to be supplied. Moreover, common sense demands that an honest professional opinion be rendered in any given case, and not a personal commercial one. Is it not evident that the vulcanite denture must necessarily be the logical reliance of the large majority of dentists, for all restorations.

While it is foreign to our subject it is opportune to make a few remarks concerning vulcanite.

Contrary to the popular opinion with both the profession and the public, well vulcanized vulcanite is not pervious to moisture and bacteria. However, there is a property of vulcanite that is not well understood by the profession that is a serious one. Rubber expands under heat until the vulcanizing temperature is attained and then shrinks as long as it remains under vulcanizing temperature (248° F.). This creates a space between the porcelain and the base plate, also between vulcanite and a metal base plate, when used, for the accumulation of filth. The diatoric teeth are much worse in this respect than the pin teeth. Furthermore, the mucous secretions with the accompanying bacteria may be strongly attached to the surface of a vlucanite denture. The patient can remove this accumulation from the exposed surface of vulcanite by the use of a stiff brush and kitchen sapolio, afterward rubbing with a cloth until the vulcanite is smooth and glossed.

The third moral or ethical proposition proposed was: "The qualifications and limitations of the dentist."

The dentist is supposed to and should qualify himself to render a reasonable service in any operation that he may attempt to perform. All men are limited in their abilities. Happy is the man who has learned his limitations and has the moral strength to govern his life accordingly.—*Pacific Dental Gazette.*

